# Reconstruction and Analysis of Cattle Metabolic Networks in Normal and Acidosis Rumen Tissue

**DOI:** 10.3390/ani10030469

**Published:** 2020-03-11

**Authors:** Maryam Gholizadeh, Jamal Fayazi, Yazdan Asgari, Hakimeh Zali, Lars Kaderali

**Affiliations:** 1 Department of Animal Science, Faculty of Animal Science and Food Technology, Agricultural Sciences and Natural Resources University of Khuzestan, Mollasani, Ahvaz 6341773637, Iran; ma.gholizade@gmail.com; 2 Department of Medical Biotechnology, School of Advanced Technologies in Medicine, Tehran University of Medical Sciences, Tehran 1416753955, Iran; Yasgari@tums.ac.ir; 3 School of Advanced Technologies in Medicine, Shahid Beheshti University of Medical Sciences, Tehran 1416753955, Iran; Hakimehzali@gmail.com; 4 Institute of Bioinformatics, University Medicine Greifswald, Felix-Hausdorff-Str. 8, 17475 Greifswald, Germany; lars.kaderali@uni-greifswald.de

**Keywords:** bovine ruminal acidosis, transcriptome profile, metabolic network

## Abstract

**Simple Summary:**

Economics of feedlot beef production dictate that beef cattle must gain weight at their maximum potential rate; this involves getting them quickly onto a full feed of high fermentable diet which can induce the ruminal acidosis disease. The molecular host mechanisms that occur as a response to the acidosis, are mostly unknown. For answering this question, the rumen epithelial transcriptome in acidosis and control fattening steers were obtained. By RNA sequencing we found the different expression profiles of genes in normal and acidosis induced steers. Then we constructed two metabolic networks for normal and acidosis tissue based on gene expression profile. Our results suggest that rapid shifts to diets rich in fermentable carbohydrates cause an increased concentration of ruminal volatile fatty acids (VFA) and toxins and significant changes in transcriptome profiles and metabolites of rumen epithelial tissue, with negative effects on economic consequences of poor performance and animal health.

**Abstract:**

The objective of this study was to develop a system-level understanding of acidosis biology. Therefore, the genes expression differences between the normal and acidosis rumen epithelial tissues were first examined using the RNA-seq data in order to understand the molecular mechanisms involved in the disease and then their corresponding metabolic networks constructed. A number of 1074 genes, 978 isoforms, 1049 transcription start sites (TSS), 998 coding DNA sequence (CDS) and 2 promoters were identified being differentially expressed in the rumen tissue between the normal and acidosis samples (*p* < 0.05). The functional analysis of 627 up-regulated genes revealed their involvement in ion transmembrane transport, filament organization, regulation of cell adhesion, regulation of the actin cytoskeleton, ATP binding, glucose transmembrane transporter activity, carbohydrate binding, growth factor binding and cAMP metabolic process. Additionally, 111 differentially expressed enzymes were identified between the rumen epithelial tissue of the normal and acidosis steers with 46 up-regulated and 65 down-regulated ones in the acidosis group. The pathways and reactions analyses associated with the up-regulated enzymes indicate that most of these enzymes are involved in the fatty acid metabolism, biosynthesis of amino acids, pyruvate and carbon metabolism while most of the down-regulated ones are involved in purine and pyrimidine, vitamin B6 and antibiotics metabolisms. The degree distribution of both metabolic networks follows a power-law one, hence displaying a scale-free property. The top 15 hub metabolites were determined in the acidosis metabolic network with most of them involved in the fatty acid oxidation, VFA biosynthesis, amino acid biogenesis and glutathione metabolism which plays an important role in the stress condition. The limitations of this study were low number of animals and using only epithelial tissue (ventral sac) for RNA-seq.

## 1. Introduction

In ruminant animals, such as cattle, healthy rumen plays an important role in production and economic efficiency. To meet the global demand for meat consumption, improving the production efficiency of cattle, would result in economic benefit. Economics of the feedlot meat production, dictates that cattle must gain weight at their maximum potential rate. This requires using intensive feeding strategies such as high energy as well as fermentable diets [1,2]. Economics also favor the processing of grain to increase the digestibility of starch. All of these factors set the stage for grain overload in the feedlot cattle and may induce digestive disorders leading to ruminal acidosis. The most common and primary form of ruminal acidosis in beef cattle fed high fermentable diets are thought to be lactic acidosis or acute acidosis [3,4]. Acute ruminal acidosis is often associated with an increase in lactic acid amount which yields pH depression and causes damage to the rumen epithelium as a consequence. Acidosis has a wide variety of clinical manifestations including the depressed feed intake, liver abscesses, diarrhea, inflammation and increases the morbidity and mortality of the stock [5,6,7]. 

The negative economic consequences resulted from the poor performance and animal health caused by acidosis, have made it one of the most prevalent animal welfare issues in the beef cattle industry. System level understanding of the bovine metabolism and relationship with their products is essential in cow breeding. However, several in vivo studies have demonstrated changes in some genes expression within the rumen tissue in response to the acidosis, but the functional and molecular host mechanisms are mostly unknown [8,9,10]. 

Systems biology is a field of biological science that aims to systematically study all the molecules and their interactions within an organism, tissue or living cell as a network. The metabolic network is a central area in systems biology and belongs to one of the best-studied biochemical networks [11]. The metabolic network is composed of small biomolecules (metabolites) and proteins (enzymes) that interact with them in order to catalyze a biochemical reaction. In fact, the goal of metabolic network reconstruction (MNR) is to identify the series metabolites involved in the biochemical reactions that define a cell metabolism [9]. 

Previous studies assayed gene expression changes in rumen epithelium under high grain concentration diets using microarray data or by PCR [3,4]. Li and coauthors studied the transcriptome changes in rumen tissue of young calves with feed induced acidosis using RNA-seq. Results from this study indicated that a starch-rich, diet-induced ruminal acidosis is accompanied by significant rumen epithelial transcriptome changes [7]. In this study we induced ruminal acidosis by feeding a starch-rich diet to beef cattle to analyze rumen epithelial tissue transcriptome. Furthermore, reconstruction of the metabolic networks of rumen epithelial tissue combined with systems biological analysis by implementing the advanced bioinformatics tools were carried out. The ventral sac of the rumen was chosen to assay changes as rumen is the first organ facing acidosis and the ventral sac has the highest capillary blood flow per unit weight mucosa of any location within the rumen [12]. The primary metabolic network in the bovine rumen tissue was reconstructed using the available bovine genome annotation and expression information. Moreover, acidosis network (subnetwork) reconstructed by mapping of differentially expressed genes (DEGs) were identified by rumen papillae tissue RNA-seq analysis with the primary network. Here we present a study that assays global transcriptional changes using RNA-seq based analysis that may contribute to enhance the understanding of ruminal acidosis. We hypothesize that the rumen tissue in acidosis cases has a more significant expression of genes involved in rapid structural changes to maintain hemostasis, nutrient absorption and energetic metabolism.

## 2. Materials and Methods 

### 2.1. Data Collection and Pretreatment 

Available genome annotation and expression information of a bovine’s tissue-specific file was downloaded from the FTP site of the UniGene database (ftp://ftp.ncbi.nih.gov/repository/UniGene/) [13]. A total of 4159 genes expressed in the rumen tissue were selected from the downloaded files (Table S1). Each gene was then queried in the Uniprot database (http://www.ebi.uniprot.org/index.shtml) to verify whether it encodes an enzyme. At this step 654 enzyme encoding genes were detected. Since the ordinary organism-specific data have many errors and does not have complete enzymes and reaction information, the KEGG bovine’s pathway information files were not directly used for the metabolic network construction [14,15]. The enzyme commission (EC) number of each gene was obtained from Uniprot [16], and the corresponding reaction for each enzyme (all substrates and products) and other information were queried in the KEGG (http://www.genome.jp/kegg/) and BRENDA (http://www.brenda-enzymes.org) databases and checked in Uniprot as well. Some genes coding the enzyme, such as endopeptidase, tryptase or protein disulfide isomerase have no definite reactions. Therefore, these enzymes were removed from the data set.

Ethical Approval: All applicable international, national, and/or institutional guidelines for the care and use of animals were followed.

### 2.2. Reconstruction and Topological Analysis of the Metabolic Networks 

In order to complete the data set for MNR, several features of metabolites were considered. These features include the component names (metabolic description, KEGG, ChEBI and PubChem identifier), chemical formula and cellular compartment [2,17]. Metabolites names usually differ between databases and there is no universal identifier between them. For this reason, they are referred by their KEGG, ChEBI and PubChem ID. However, the formula of a metabolite used for matching metabolites, is not usable as a unique identifier. Two different metabolites aspects of function can have the same formula because there are various structures of atoms. The common small molecules (known as the currency metabolites such as ATP, NADH, H_2_O, CO_2_, etc.) were not removed from the network representation. A number of 15 compartments were assayed accounting for 1771 reactions and 1429 metabolites. The final form of data was converted into a Systems Biology Markup Language (SBML) file format [18] in order to be visualized with Cytoscape software (vs. 3.6.1). For the acidosis model, we have used RNA-Seq data in order to perform a mapping into the normal model. Hence, the differentially expressed genes have been mapped into the normal network to build an acidosis state. Again, the final network has been created in the SBML file format. Some topological properties of the reconstructed networks have been also analyzed using Network Analyzer and cytoHubba plugins in Cytoscape [19,20]. The node degree distribution is one of the most critical topological parameters that gives the number of nodes with a degree (link) k (for k = 0, 1, …) that is defined as:

*P(k)* ~ *k*^-γ^ (1)

where P(k) determines the variation in the nearest neighbors (degree distribution) and γ determines many properties of the system [13]. Real networks display a scale-free property therefore, the degree of a real network follows a power-law distribution. In the scale-free network most nodes have only a few links, but a few nodes (called hubs) have a very large number of links. P(k) for a scale-free network has no well-defined peak, and for large k it decays as a power-law P(k) ~ k^-γ^ appearing as a straight line with slope -γ on a log-log plot [21,22]. The shortest path length for nodes n and m define as L (n, m), which determines the number of edges that have to be crossed between n and m. For the whole network, the mean path length represents the average of the shortest paths among all pairs of nodes that is also known as the characteristic path length and offers the expected distance between n and m [20]. On the other hand, in this investigation, we first identified DEGs between control and acidosis tissue by rumen papillae tissue RNA-seq analysis and then these DEGs mapped to the primary network to construct the acidosis metabolic network (subnetwork) using bottleneck algorithm and double screening scheme.

### 2.3. Animal Management and Rumen Tissue Collection 

Six Holstein steers (230 days old) born in spring 2015, were randomly divided into two groups (control and acidosis) and managed in the Agricultural resources farm of the University of Tehran. The high fermentable diet to induce acidosis was fed for 130 days being composed of 10.5% alfalfa hay, 14.5% corn silage, 34% barley grain, 23.3% corn grain ground, 9% soybean meal, 3% wheat bran, 2.5% rice bran and 3.2% supplement. The control diet was composed of 10.5% alfalfa hay, 14.5% corn silage, 34% barley grain, 5.3% corn grain ground, 9% soybean meal, 10.5% wheat bran, 6% rice bran, 5% sucrose, 2.5% fat powder and 3.2% supplement [3,8]. The final average body weight before slaughter for the acidosis and control groups was 529 ± 20 and 570 ± 10 kg, respectively. Clinical signs of acidosis were observed in all three animals. The ruminal pH was measured in the 1st, 30th and 129th days by manually inserting a pH probe into the rumen through a cannula and average data over 6 time-points in a single day (−8, −4, 0, 2, 4 and 8 h relative to grain feeding) (Table 1). After slaughter, for each sample, a 4 cm^2^ piece of rumen tissue was obtained from the central region of the ventral sac and rinsed with sterilized PBS buffer (pH = 6.8) before being placed in a 50 mL tube containing RNA later solution (Invitrogen, Carlsbad, CA, USA). The samples were then stored at −80 °C until further processing. The ventral sac of the rumen was chosen as sampling site because it has the highest capillary blood flow per unit weight mucosa of any location within the rumen [12].

### 2.4. RNA Extraction, Quantification and Sequencing 

Total RNA was extracted from the tissue samples using Trizol Reagent kit (Invitrogen, Carlsbad, CA, USA) according to the manufacturer’s protocol. The RNA integrity of the total RNA samples was assessed using the Agilent 2100 Bioanalyzer (Agilent Technologies, Santa Clara, CA, USA). Only samples with RNA integrity greater than 7 were subjected to RNA-seq library construction. Nanodrop 2000c spectrophotometer (Thermo Scientific, Wilmington, DE, USA) was used to measure the concentration and purity. RNA samples were stored at −80 °C until RNA-seq library preparation. RNA-sequencing library preparation was done using Illumina TruSeq ribo-zero gold kit following the manufacturer’s instructions. For each sample, a cDNA library was generated from 100 ng of total RNA. mRNA was enriched by removing rRNA from the total RNA with Ribo-Zero TM Magnetic Kit. mRNA was fragmented into short fragments (about 200–700 bp), then positive-strand cDNA was synthesized by random hexamer primers using the fragments as templates. Buffer, dNTPS, RNase H and DNA polymerase *I* was added to synthesize the negative-strand cDNA. The double stranded cDNA was purified using the QiaQuick PCR extraction kit and then used for end-polishing. Sequencing adapters were ligated to fragments, then the second strand was degraded using UNG (Uracil-N-Glyosylase). The fragments were purified by agarose gel electrophoresis and enriched by PCR amplification. Sequencing was performed on Illumina HiSeqTM 2000 to obtain high quality, 100 bp paired-end reads. 

### 2.5. Sequence Data Processing and Differential Gene Expression Analysis

The Tuxedo pipeline in the statistical environment R version 3.4.4, has been used for the RNA-seq data analysis. The pipeline consists of TopHat, [23] Cufflinks (v. 2.2.1) and cummeRbund (v. 3.4) software. Sequencing quality was assessed using FastQC (v. 0.11.2). The data were preprocessed using Trimmomatic (v.0.32) to filter out low-quality reads, remove adaptors and trim low-quality regions of reads with default parameters [24]. The transcriptome mapping and alignment of the reads to the bovine reference genome (ARS-UCD1.2) were performed using Bowtie2 (v. 2.0.1) and TopHat2 (v. 2.0.9) with default parameters, resulting into BAM format files [25]. Cufflinks was also used to assemble transcripts and estimate their abundance as Fragments Per Kilobase of transcript per Million mapped reads (FPKM, cut-off ≥ 1), which normalizes transcript expression for transcript length and the total number of reads per sample. All assemblies were merged into a single transcriptome annotation using Cuffmerge (v. 2.2.1). Cuffdiff (v. 2.2.1.5), as a part of the Cufflinks package, was used to identify the differentially expressed genes (*p* < 0.05). The list of significant genes was further screened using PANTHER for further functional annotation. Results were visualized using the cummeRbund R library. Then the differentially expressed genes of this step of data analysis used for mapping and reconstruction of an acidosis metabolic network (subnetwork) using a bottleneck algorithm and double screening scheme. Bottleneck index calculates the number of shortest paths going through a certain node. Therefore, nodes with the highest bottleneck index, represent the critical points of the network. In addition, they could also control most of the information flow in the network [26]. 

The ssizeRNA package of R [27] was used with 71 million clean reads and 2 fold changes in average, the dispersion parameter for each gene equals 0.1 and 10 simulations. The result was 0.86 for the power of test and 0.04 for the false discovery rate. 

### 2.6. Quantitative Real-Time PCR (qRT-PCR) Validation of Selected Differentially Expressed Genes

The expression of four randomly selected DEGs related to butyrate metabolism (OXCT1), the CoA biosynthesis pathway (DPYD), fatty acid oxidation (CPT1A), and the pantothenate biosynthesis pathway (FOXF1) was detected using RT-qPCR to confirm their differential expression between acidosis and control groups. Primers targeting four selected DEGs were designed using Primer-BLAST (NCBI, Bethesda, MD), Primer Express software (Applied Biosystems, Foster City, CA). The specificity of primers was checked with BLAST (NCBI) and the UCSC In-Silico PCR program (Table S2). The RT-qPCR reactions were performed with SYBR Green (Fast SYBR® Green Master Mix; Applied Biosystems) using StepOnePlus™ Real-Time PCR System (Applied Biosystems, Foster City, CA, USA) with the fast cycle and the following program: 20 s pre-denaturalization at 95 °C, followed by 40 cycles of 3 s denaturation at 95 °C and 30 s annealing and extension at 60 °C. Gene expression values were normalized to the reference gene of β-actin in the same sample. The relative changes in each gene expression were calculated using the 2^−ΔΔCT^ (cycle threshold, CT) method.

## 3. Results

### 3.1. The Primary Metabolic Network 

The primary network was a directed metabolic-centric network that reconstructed for 15 compartments including the cytoplasm, nucleus, cytosol, Golgi apparatus, mitochondrion, membrane protein, endoplasmic reticulum, peroxisome, plasma membrane, lysosome, extracellular exosome, cytoskeleton, cell membrane, extracellular exosome and mitochondrial membrane. The network includes 1429 metabolites as the nodes and 535 genes catalyzing 1771 relevant reactions as the edges. The topological network parameters of the primary metabolic network are listed in Table 2. The network data file is attached to Table S3.

**Figure 1:**
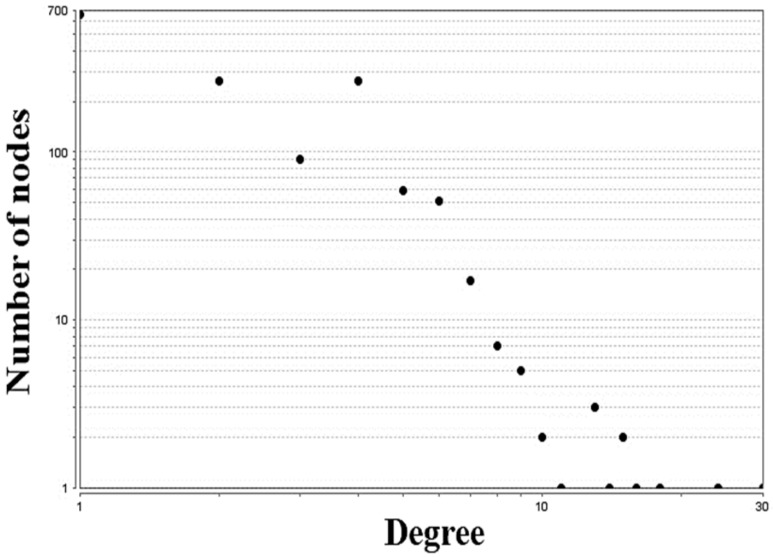
Topological properties of the reconstructed network for healthy ruminal epithelium. Node degree distribution.

Node degree distribution of the primary network is plotted in Figure 1. The degree distribution of a real network follows a power-law distribution. In the present study, the correlation coefficient (R) is 0.91 (*p* < 0.0001), and γ is taken as 0.89. The small value of γ implies that the role of the hub nodes is more important in the network. In the networks with a power-law degree distribution, the majority of the nodes have only one or two neighbors while coexisting with many nodes with hundreds and some even with thousands of neighbors. For these networks there exists no typical node, and they are therefore often referred to as scale-free. Unlike exponential networks, scale-free networks are extremely heterogeneous, with their topology being dominated by a few highly connected nodes (hubs) which link the rest of the less connected nodes to the system. In this respect the emergence of power-law distribution is intimately linked to the growth of the network in which new nodes are preferentially attached to already established nodes, a property that is also thought to characterize the evolution of biological systems. Our results convincingly indicate the probability that a given substrate participates in k reactions follows a power-law distribution; in other words, metabolic networks belong to the class of scale-free networks. The shortest path length is one of the most critical topological parameters, also called the distance between two nodes [20]. The average path length in the present reconstruction is equal to 13.142. Radrich et al. (2010) demonstrated the value of the average path length becomes significantly higher when common small molecules are removed from the network [28]. The network diameter is the largest distance between the two nodes whose value in the present network is as long as 38. The results of the centrality analysis using cytoHubba plugin, have been provided in Ma and Zeng (2003) [17]. As it was expected, the top 20 nodes are the currency metabolites usually found in all metabolic networks. Removing the currency metabolites, the top 15 nodes with the highest degree centrality indexes are presented in Table 3.

Since the existing network is scale-free and hubs play a significant role in maintaining the network and system biology of the rumen epithelium tissue, assays were conducted to identify their functions [13]. The centralization for the present network is estimated as 0.119 which is given by *n/(n-2) (max(k)/(n-1)- Density*. Centralization is a measure of how a network is focused around central nodes or assesses if a network has star-like topology. This value is close to 0 for a decentralized network and approaches 1 for the network resembling a star. The value of heterogeneity (defined as the coefficient of variance of the connectivity) in our network is close to 0.862 which indicates that some metabolites are highly connected and most of them have a lesser number of connections.

### 3.2. Transcriptome Profiling of the Rumen Epithelium 

**Figure 2:**
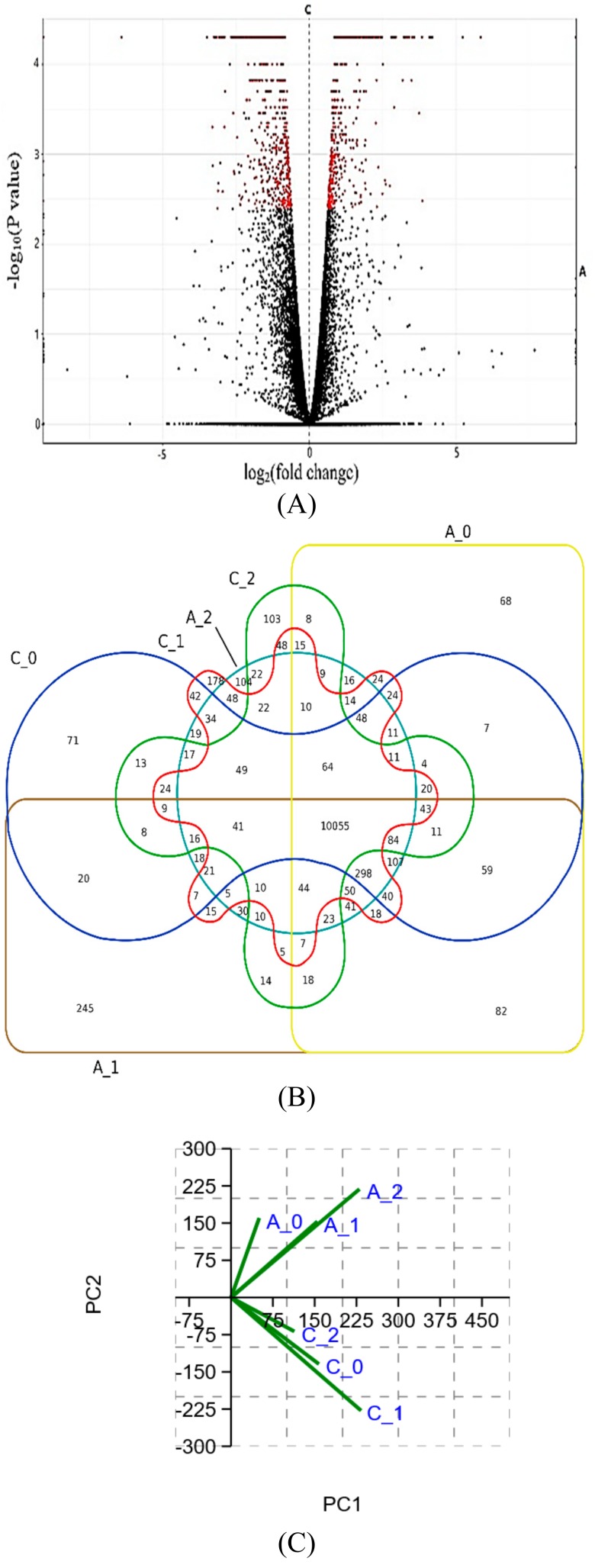
**(A)** Volcano plot is representing the relationship between fold-change and significance. Red points identified differentially expressed genes between control and acidosis samples. We identified 1074 differentially expressed genes (*p* < 0.05), of which 627 were up-regulated and 447 genes were down-regulated in the acidosis group. **(B)** InteractiVenn plot to identify similarities and different gene expressions between each sample (control samples: C_0, C_1, C_2 and acidosis samples: A_0, A_1, A_2). **(C)** PCA plot shows that gene expression profiles of normal and acidosis tissue fall into distinct clusters.

The average of clean reads (after removing adaptor sequence and low-quality reads) was 71 million reads with an average of 70,975,460 ± 272,875 reads in normal samples and 71,142,189 ± 474,023 reads in the acidosis samples. The average of Q20 and GC percent were 97.96% and 50.87%, respectively (Table S4). The total number of expressed genes (genes with at least one read in every sample) for the rumen tissue was 24,494, and the overall read alignment rate to the bovine reference genome (ARS-UCD1.2) was evaluated as 87% ± 3.43%. A number of 1074 DEGs were identified in the rumen tissue between the normal and acidosis samples using the FDR-corrected p-value with a significance level of α =0.05 (Table S5). Then, DEGs mapped to the primary network to reconstruct the acidosis metabolic network. Further validation of the differential expression using reverse transcription quantitative real-time PCR (RT-qPCR) showed that the expression of OXCT1, DPYD, CPT1A and FOXF1 was consistent as detected with the RNA-seq data (Table S2). Additionally, we identified 978 isoforms (up-regulate: 584, down-regulate: 394), 1049 transcription start sites (TSS) (635 up-regulated and 414 down-regulated), coding DNA sequence (CDS) (607 up-regulated and 391 down-regulated) and 2 promoters (up-regulated) have differentially expressed in the rumen tissue between the normal and acidosis samples. Of the differentially expressed genes (DE), 627 were up-regulated, and 447 were down-regulated in acidosis. The functional analysis using PANTHER [29], indicated that the down-regulated genes control the biological processes such as; Biological regulation (GO: 0065007), Cellular process (GO: 0009987), Localization (GO: 0051179), Locomotion (GO: 0040011), Metabolic process (GO: 0008152), Multicellular organismal process (GO: 0032501), Response to stimulus (GO: 0050896). Conversely, PANTHER analysis for the up-regulated genes, illustrated that these genes involved in the biological adhesion (GO: 0022610), Biological regulation (GO: 065007), Cellular component organization (GO: 0071840), Developmental process (GO: 0032502), Immune system process (GO: 0002376), Multicellular organismal process (GO: 0032501), and Reproduction (GO: 0000003) (Table S6). There were 111 enzymes identified which presented significant changes in the expression under the acidosis condition among which, 46 enzymes were up-regulated and the remaining 65 down-regulated. The most significant up-regulated and down-regulated enzymes are presented in Table 4 and Table 5. The PCA and volcano plots depict the global gene expression dynamics between the rumen epithelia of normal and acidosis steers. Figure 2A shows a volcano plot of the present data, depicting -log10 p-values over log2fold changes. The significantly differentially expressed genes are illustrated as red dots. The PCA plot (Figure 2C) shows that the gene expression profiles of the normal and acidosis tissues fall into distinct clusters. The result of cufflinks is converted to the InteractiVenn plot in order to identify the similarities and different genes expression between each sample group individually (Figure 2B). 

### 3.3. Acidosis Metabolic Network

**Figure 3:**
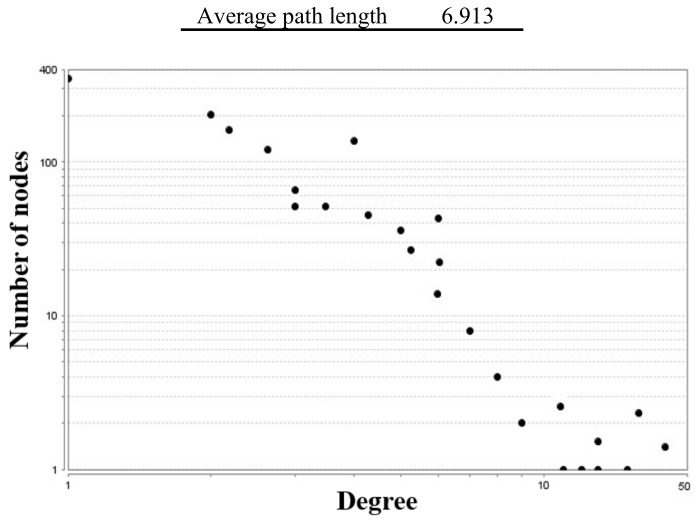
Node degree distribution of reconstructed metabolic network for acidosis ruminal epithelium.

**Figure 4:**
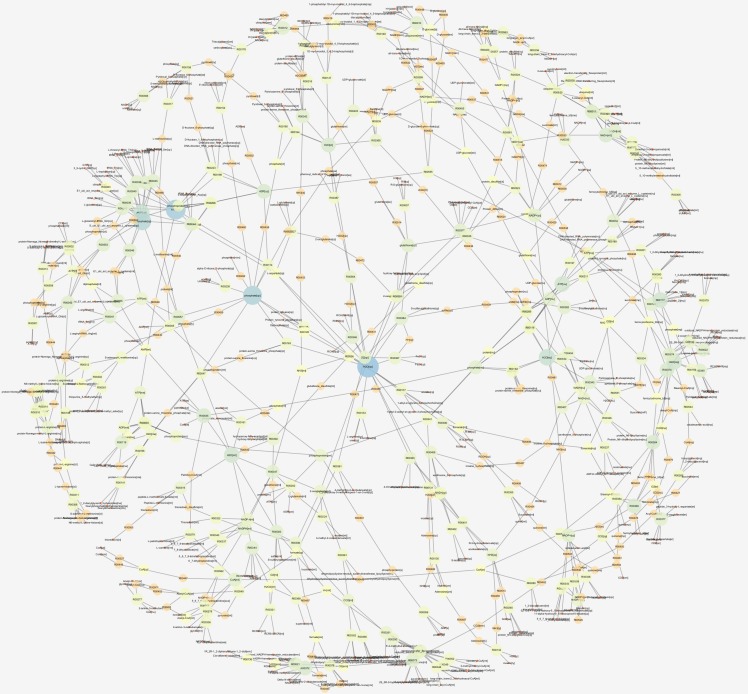
The reconstructed acidosis metabolic network for bovine.

The acidosis metabolic network (as a subnetwork) based on the bottleneck and double screening scheme methods reconstruction which included 832 metabolites and 1021 reactions (Table S7). Node degree distribution of the network is plotted in Figure 3 and the topological network parameters are given in Table 6. The top 15 hubs identified by the bottleneck (BN) algorithm and double screening scheme (DSS) methods are shown in Table 7. Figure 4 depicts the graphical acidosis metabolic network representation. The search results in the KEGG pathway clarified that stearoyl-CoA, 2-oxoglutarate and lactate are intermediates in the fatty acid metabolism pathway (map01212), citrate cycle (map00020) and glycolysis (map00010), respectively and acetyl-CoA is a metabolite linking between the citrate cycle, fatty acid pathway and glycolysis. 

To reconstruct the metabolic networks, we used a graph theory- based method that metabolites correspond to the nodes and reactions correspond to the edges as a metabolite-centric network. The existing networks were undirected because all the reactions were reversible and reconstructed for 15 compartments that most of the hub metabolites were in mitochondrion, cytoplasm, and nucleus. The small-world property, is one of the most critical real network properties. The networks with this property have a characteristic feature of regional specialization and relatively efficient information transfer [30]. This property characterized by a diameter network, which is defined as the average minimum path length between all pairs of nodes. The value of the diameter in the primary and subnetwork were 38 and 28, respectively. Embar et al (2016) reported the path length is of biological interest because it is assumed that a functionally related gene would be more closely connected in the metabolome in a gene network [31]. The average path length presents a measure of the network’s overall navigability and it is assumed that functionally related genes would be more closely connected in the metabolome that the value in the primary and subnetwork which were equal to 13.142 and 6.913, respectively. Zhang et al. (2005) demonstrated that the diameter in all 13 real networks examined (biological, social, technological and linguistic), is higher than the random expectation [32]. Ma and Zeng (2003) revealed that the average path length and diameter have greater value in eukaryotes (9.75 and 33.1) than bacteria (7.23 and 20.6) [17]. Our results of topological analysis networks showed these two crucial parameters were nearer to the values of eukaryotes and far off bacteria.

## 4. Discussion

The profound understanding of the bovine rumen epithelium metabolism and changes during the ruminal acidosis have a tangible effect on cow breeding and provide crucial biological information. Ruminal acidosis is one of the most important disorders and production problems in the beef industry occurred by low ruminal pH in the range of 5.2 and 6 for a prolonged period [5]. Ruminal pH depends on the Henderson-Hasselbach balance that is defined as [9]:

pH = pKa + log [(acid^−^) (H acid)^−1^] (2)

During the microbial ruminal fermentation, the population of microorganisms (bacteria, ciliate protozoa, anaerobic fungi and bacteriophages) ferments the carbohydrates and protein to the short-term intermediates such as sugars and amino acids [9]. The products of this initial degradation are metabolized to produce energy, methane, carbon dioxide, heat and volatile fatty acids (VFA). At the physiological pH of rumen fluid, 90–99% of the acid is in the dissociated form as a base conjugate (VFA-) and H+ that are absorbed through the rumen wall [33]. 

When cattle are fed with a high level of rapidly digestible carbohydrate to gain weight at their maximum level, VFA production exceeds the rumen acids absorption capacity which causes accumulated VFA and dropping ruminal pH. Lactic acid has a lower pKa (3.80) than other VFA, and its concentration is low during the ruminal acidosis. However, as the pH drops below 5.7, the rate of lactic acid formation exceeds the absorption capacity which causes a drastic decrease in pH value [34]. If this condition continues, the lactic acid entry into the blood system causes a big challenge in metabolism, changes the population of microorganisms, rumen motility, and the systemic body fluid balance. Recent management practices strive to minimize the ruminal acidosis occurrence, but the need for a better understanding of the disease’s etiology, prevention and treatment are still felt [5,6]. 

The main goal of this study was the development of a system-level understanding of biological changes in rumen tissue under acidosis. For this purpose, the focus has been on the reconstruction of a bovine rumen tissue-specific network using the transcriptome data of bovines with and without acidosis in order to compare the acidosis-related metabolites and reactions based on the network topology. Studies conducted on the acidosis using the metabolic network method can provide new information about it through the identification of the novel metabolic function and metabolite biomarkers. Because a change in the concentration of one metabolite, leads to the change of several cascade reaction pathways that may yield the diverse phenotypes and disorders [35]. 

Of a total of 1074 DEGs, 111 genes encoded an enzyme. The analysis of pathway and reaction associated with up-regulated enzymes, indicated that most of these enzymes are involved in 3 central metabolic pathways including the fatty acid metabolism, biosynthesis of amino acids, pyruvate and carbon metabolism (Table 8). The map profiling results showed the significant up expression of the OXCT1 gene encoding succinyl-CoA transferase (2.8.3.5) under the acidosis. This enzyme takes part in butyrate metabolism within the mitochondrion and also the nucleus in order to produce butyrate as the microbial ferment product of a high starch diet and as one of the important substrates in the ruminant energy metabolism. Hernández et al. (2014) reported a significant increase in the rumen VFA concentration (150–225 mM) of the caws under ruminal acidosis [6]. Previous studies have reported that in acidosis lipopolysaccharide (LPS) derived from the rumen down-regulates stearoyl-CoA desaturase 1 expression (SCD1) and demonstrated up-regulation of the genes involved fatty acid β-oxidation [10]. The present results of the gene expression profile assay indicated the obvious pattern as up-regulation of the genes CPT1A, ALDH9A1and TSPAN12, associated with the fatty acid oxidation and down-regulation of SCD (stearoyl-CoA 9-desaturase) in the acidosis group.

The SCD enzyme is responsible for forming a double bond in Stearoyl-CoA and plays an important role in fatty acid biosynthesis, regulating the genes expression which is involved in the lipogenesis and also regulating the mitochondrial fatty acid oxidation [36]. Stearoyl-CoA is a long-chain acyl CoA ester that acts as an intermediate metabolite in the biosynthesis of the monounsaturated fatty acids. This oxidative reaction is catalyzed by the iron-containing, microsomal enzyme and SCD. NADH supplies the reducing equivalents for the reaction, the flavoprotein is cytochrome b5-reductase and the electron carrier is the heme protein cytochrome b5. The result of analysis using cytoHubba plugin show Stearoyl-CoA (C00412) and ferrocytochrome b5 (C00996) are hub metabolites in the subnetwork. Hubs in scale-free networks have a key biological role and significant effect in maintaining the network [13]. For this reason, improving the bovine acidosis researches seem to be useful. PDK2 (5.3.99.4), PTGDS (5.3.99.2), and TNS2 (1.11.1.9) take part in the arachidonic acid metabolism, whilst PLA2G4A (3.1.1.4) and TSPAN12 (1.14.14.1) participate in both arachidonic (ec00590) and linoleic acid (ec00591) metabolisms. Linoleic (18:2 Δ9, 12) and arachidonic acids (20:4 Δ5, 8, 11, 14) are essential long-chain polyunsaturated fatty acids (LC-PUFAs) and a type of linoleic acid (ω-6) can be converted to the arachidonic one [37]. In addition, both of them have important biological functions as a component of phospholipid membranes and arachidonic acid is also present in cell membranes of erythrocytes, neutrophils and monocytes.

The analysis results of the DEGs illustrated significant high expression of FOXF1 (2.6.1.42) and DPYD (1.3.1.2) involved in the Pantothenate and CoA biosynthesis pathway (ec00770). Pantothenate (vitamin B5) is the key precursor for the biosynthesis of coenzyme A [36]. This vitamin is a carrier protein, having a phosphopantetheine group that shuttles intermediates between the active sites of enzymes involved in the fatty acid and non-ribosomal peptide synthesis. Additionally, the results of the sub-network analysis showed that acetyl-CoA (C00024) and S-adenosyl-L-methionine (C00019) cytoplasmic are hub metabolites. The conversion of pyruvate into acetyl-CoA is a crucial step in the carbon energy metabolism that occurs by pyruvate formate-lyase (PFL; 2.3.1.54) or pyruvate ferredoxin oxidoreductase (PFO;1.2.7.1). Pyruvate formate-lyase comprises two cofactors of S-adenosyl methionine and thiamine diphosphate. The pathways results indicated that the biosynthesis of amino acids in the ruminal acidosis increases which might lead to the increased rumen pyruvate and VFA content in the acidosis condition [38]. In the rumen metabolism pyruvate and isobutyrate for valine, acetate for serine, propionate for arginine and histidine, butanoic acid for leucine and 2-methyl butyrate for isoleucine are the main substrates and precursors for the biogenesis of these amino acids. The result of GC–MS analysis by Xue et al. (2018) showed that the SARA-inducing diet significantly increases the propionate, pyruvate, lactate, glycine and valine contents in the rumen of dairy cows compare to the control one [10]. The results of pathways analysis indicated that TNS2 (glutathione peroxidase; 1.11.1.9) and GSTM3 (glutathione transferase; 2.5.1.18) participate in the glutathione metabolism (ec00480) and glutathione (C00051) was identified here as a hub metabolite in the subnetwork. Glutathione is a non-protein thiol for protecting cells against the oxidative stress condition via detoxification of the oxygen-derived free radicals using glutathione peroxidase (GSH-Px) and transferase enzymes [39]. These enzymes are antioxidants and play an important role in stress conditions such as immune and inflammatory response. Xue et al. (2018) detected significant differences in the glutathione metabolism of bovine rumen tissue between the control and SARA groups [10]. Also, Jiang et al. (2014) reported that glutathione transferase is significantly up-regulated in the goat liver tissue of the SARA group compared to the control one [8]. 

The pathway analysis of the down-regulated enzymes showed that most of these enzymes are involved in the purine and pyrimidine, vitamin B6 and biosynthesis of the antibiotic’s metabolisms. Purine and pyrimidine are basic components of DNA and RNA in all living cells that participate in the wide range of biological processes. Purine and pyrimidine metabolism include the synthesis and degradation of these components with deferent enzymes and metabolites [40]. ADA (3.5.4.4: adenosine deaminase), PNP (2.4.2.1: purine-nucleoside phosphorylase) and ENPP4 (3.6.1.29: bis(5′-adenosyl)-triphosphatase) are involved in the purine metabolism pathway (ec00230). Also, UPP1 (2.4.2.3: uridine phosphorylase) and CTPS1 (6.3.4.2: CTP synthase) are involved in the pyrimidine metabolism pathway (ec00240). With the reduced expression of these enzymes, the appropriate substrates and other alternative products are accumulated (dATP and dGTP) and this leads to the toxic effects on the cells of the immune system. Cytidine triphosphate synthase (CTPS) catalyzes the last step in the pyrimidine biosynthesis which is the irreversible reaction of the converted uridine triphosphate (UTP) into CTP with the glutamine and ATP consumption. The results of the enzymes pathway analysis showed that the enzymes involved in the biosynthesis of antibiotics (ec01130) including ISYNA1 (5.5.1.4), SDS (4.3.1.17), ODC1 (4.1.1.17), ARG1 (3.5.3.1), PSPH (3.1.3.3), PSAT1 (2.6.1.52), TALDO1 (2.2.1.2), MTHFD2 (1.5.1.15) and PGD (1.1.1.44), have been down-regulated significantly. Antibiotics, one of the secondary metabolites, encompass a broad group of chemical compounds produced by fungi, bacteria and other organisms with a different molecular target. They have a wide range of functions that can selectively destroy or inhibit the growth of other microorganisms [41]. Sodium monensin is a polyether antibiotic produced by *Streptomyces cinnamonensis* which acts by inhibiting the growth of *Streptococcus bovis* (and other major lactic acid-producing strains except for Selenomonas) and leads to the lactate production decrement and rumen pH increment.

The pathways analysis results further indicated the down-regulation of PDXK (2.7.1.35: pyridoxal kinase) and PSAT1 (2.6.1.52: aspartate transaminase) was involved in vitamin B6 metabolism. Beef meat is a major source of B6, B12 Thiamin, Riboflavin and Niacin vitamins. Vitamin B6 is part of the vitamin B group and pyridoxal phosphate is the coenzyme form of this vitamin participating in the reactions related to the amino acid, glucose and lipid metabolism. Pyridoxal kinase (pdxK) is an essential enzyme in the terminal point of the vitamin B6 pathway which causes phosphorylation of pyridoxal to produce pyridoxal phosphate; therefore, a decreased expression of pdxK leads to vitamin B6 deficiency. Vitamin B6 deficiency causes impair weight gain, anorexia, and diarrhea which are some outward signs of an acidosis disorder.

## 5. Conclusions

In summary, this study gave a new comprehensive insight into the ruminal acidosis metabolic network. Transcriptome profiling and multivariate analysis were used to provide the metabolic network data of 6 rumen tissue Holstein cattle fed with normal and induced acidosis diets. As a first step, the normal metabolic network in the bovine rumen tissue was reconstructed using the available bovine genome annotation and expression information and then the differentially expressed genes of RNA-seq data analysis were used for mapping and reconstruction of an acidosis network. We identified the change in genes, enzymes and hub metabolites associated with acidosis in the rumen. With rapid shifts in the use of rich diets in fermentable carbohydrates, significant changes were found in the transcriptome profiles of the rumen epithelial tissue. The PCA of rumen data showed that acidosis samples are clustered closer to each other and separated from the control samples. Analysis of the pathways and reactions associated with up-regulated enzymes, showed that most of these enzymes are involved in the fatty acid metabolism, biosynthesis of amino acids, pyruvate and carbon metabolism. Additionally, most of the down-regulated enzymes are involved in the purine and pyrimidine, vitamin B6 and antibiotics metabolisms. The top 15 hub metabolites were determined in the acidosis network from which stearoyl-CoA and ferrocytochrome b5 are involved in the fatty acid oxidation, acetyl-CoA and S-adenosyl-L-methionine cytoplasmic in VFA biosynthesis, L-serine and L-arginine in amino acids biogenesis and glutathione in glutathione metabolism that play an important role in the stress condition such as the immune and inflammatory response. The network’s distribution degree follows a power-law distribution hence displays a scale-free property. The values of diameter and average path length in this network were 28 and 6.913, respectively, indicating that the present network has small world property. 

The achievements of the present study indicate that the use of a high grain diet could elicit a substantial change in the gene expression and transcriptome response in the ruminal tissue. Furthermore, the present results provide a fundamental understanding of which genes are associated with acidosis and may lead to the identification of a biomarker for selecting the best cows for animal breeding. Although significant genes were analyzed and known metabolic data collected from the different databases reconstructed the metabolic network, many factors such as CDS, promoter, TSS and isoform might affect the better interpretation and results of the system biology investigation. The limitations of this study were a low number of animals and using only epithelial tissue (ventral sac) for RNA-seq. Assaying liver and lung tissues are also suggested in order to provide more detailed information on the effects of acidosis on fattening steers. 

## Figures and Tables

**Table 1:** Ruminal pH of normal and acidosis groups.

Group	The 1st Day ^‡^	The 30th Day ^‡^	The 129th Day ^‡^
control	5.71; 5.89; 5.88	5.57; 5.97; 5.87	6.03; 6.17; 6.0
acidosis	5.95; 5.23; 5.64	5.97; 5.17; 5.57	5.19; 5.10; 5.24

‡ Each value represents the average pH of one animal.

**Table 2:** General properties of the constructed network.

Parameters	Values
Number of nodes	1429
Number of edges	1771
Network density	0.002
Network heterogeneity	0.862
Number of self-loops	0
Network diameter	38
Network centralization	0.019
Average path length	13.142
Average number of neighbors	2.476

**Table 3:** Top 15 hub metabolites of the metabolic network of bovine rumen tissue.

Metabolites	Neutral Formula	KeggID	PubChemID	Compartment
CoA	C21H36N7O16P3S	C00010	49600643	Mitochondrion
glutathione	C10H17N3O6S	C00051	3353	Cytoplasm
S-adenosyl-L-homocysteine	C14H20N6O5S	C00021	439155	Nucleus
S-adenosyl-L-methionine	C15H22N6O5S	C00019	34755	Nucleus
2-oxoglutarate	C5H6O5	C00026	3328	Mitochondrion
Acetyl-CoA	C23H38N7O17P3S	C00024	3326	Cytoplasm
D-fructose_6-phosphate	C6H13O9P	C00085	3385	Cytoplasm
ubiquinone	C19H26O4	C00399	5280346	Mitochondrion
formate	CH2O2	C00058	3358	Mitochondrion
electron-transferring flavoprotein	C27H30N9O15P2	C04253	6918	Mitochondrion
L-tryptophan	C11H12N2O2	C00078	3378	Cytoplasm
pyruvate	C3H4O3	C00022	3324	Mitochondrion
L-tyrosine	C9H11NO3	C00082	3382	Cytoplasm
tetrahydrofolate	C19H23N7O6	C00101	3401	Mitochondrion
L-serine	C3H7NO3	C00065	3365	Nucleus

**Table 4:** The top up-regulated enzyme in the bovine rumen tissue under acidosis condition.

Gene	EC Number	Enzyme Name	Reaction	*P*-Value
HSPA6	4.2.1.134	very-long-chain (3R)-3-hydroxyacyl-CoA dehydratase	R10827	5.00E-05
GUCY1B1	4.6.1.2	guanylate cyclase	R00434	0.0001
PDK2	5.3.99.4	prostacyclin synthase	R02267	0.0019
PTGDS	5.3.99.2	prostaglandin-D synthase	R02266	0.0021
OXCT1	2.8.3.5	succinyl-CoA transferase	R01780	0.0002
SLC24A3	3.1.1.34	lipoprotein lipase	R01369	0.0001
PLA2G4A	3.1.1.4	phospholipase A2	R01313	0.004
PDE5A	3.1.4.35	cGMP phosphodiesterase	R01234	5.00E-05
CYP2W1	3.1.4.17	cyclic 3′,5′-phosphodiesterase	R03259	0.00085
PLCD3	3.1.4.11	Phosphorinositidase C	R03435	0.00355
EPHX1	3.3.2.9	microsomal epoxide hydrolase	R07627	0.00085
DYSF	3.6.1.5	ATP-diphosphatase	R10443	5.00E-05
GSTM3	2.5.1.18	glutathione transferase	R03522	0.0003
FOXF1	2.6.1.42	branched-chain-amino-acid transaminase	R01090	0.0002
PFKM	2.7.1.11	6-phosphofructokinase	R00756	0.0015
CHPT1	2.7.8.2	diacylglycerol cholinephosphotransferase	R01321	0.0012
CDS2	2.7.7.41	phosphatidate cytidylyl transferase	R01799	0.00215
MMP16	2.7.7.40	CDP ribitol pyro phosphorylase	R02921	0.0027
CEP112	2.7.12.2	MAP kinase kinase	R00162	0.0014
CHRNA3	1.14.13.39	nitric-oxide synthase (NADPH)	R00557	5.00E-05
TNS2	1.11.1.9	glutathione peroxidase	R00274	0.0018
TSPAN12	1.14.14.1	flavoprotein monooxygenase	R04122	5.00E-05
DPYD	1.3.1.2	dihydrouracil dehydrogenase (NADP+)	R00978	5.00E-05
ALDH9A1	1.2.1.3	aldehyde dehydrogenase (NAD+)	R00538	0.0001
AOC3	1.4.3.21	primary-amine oxidase	R01853	0.0001
SOX5	1.4.3.1	D-aspartate oxidase	R00359	5.00E-05
MAOB	1.4.3.4	monoamine oxidase	R11354	5.00E-05
CILP	1.7.1.7	GMP reductase	R01134	5.00E-05
AASS	1.5.1.8	lysine-2-oxoglutarate reductase	R00716	5.00E-05
MTHFR	1.5.1.20	methylenetetrahydrofolate	R01224	5.00E-05
GATM	2.1.4.1	glycine amidino transferase	R00565	0.00015
SCN7A	2.1.1.43	N-methyltransferase	R03938	5.00E-05
CPT1A	2.3.1.21	Palmitoyl carnitine transferase	R01923	5.00E-05

**Table 5:** The top down-regulated enzyme in the bovine rumen tissue under acidosis condition.

Gene	EC Number	Enzyme Name	Reaction	*P*-Value
ASNS	6.3.5.4	asparagine synthase	R00578	5.00E-05
CTPS1	6.3.4.2	CTP synthase	R00573	5.00E-05
TARS	6.1.1.3	threonyl-tRNA synthetase	R03663	0.00095
WARS	6.1.1.2	tryptophanyl-tRNA synthetase	R03664	0.0013
YARS	6.1.1.1	tyrosine-tRNA ligase	R02918	0.0003
ISYNA1	5.5.1.4	inositol-3-phosphate synthase	R07324	0.0005
CTH	4.4.1.1	cystathionine gamma-lyase	R01001	5.00E-05
SDS	4.3.1.17	L-serine ammonia-lyase	R00220	5.00E-05
CA3	4.2.1.1	carbonate dehydratase	R00132	0.00075
ODC1	4.1.1.17	ornithine decarboxylase	R00670	5.00E-05
ENPP4	3.6.1.29	bis(5′-adenosyl)-triphosphatase	R00187	0.0019
PPA1	3.6.1.1	inorganic diphosphatase	R00004	0.0001
ADA	3.5.4.4	adenosine deaminase	R01560	5.00E-05
ARG1	3.5.3.1	arginase	R00551	5.00E-05
GGH	3.4.19.9	folate gamma-glutamyl hydrolase	R04242	0.0033
PSPH	3.1.3.3	phosphoserine phosphatase	R00582	0.0003
PRDX6	3.1.1.4	phospholipase A2	R01313	0.00115
ABHD12	3.1.1.23	acyl glycerol lipase	R01351	0.0003
PDXK	2.7.1.35	pyridoxal kinase	R00174	0.0034
GK	2.7.1.30	glycerol kinase	R00847	5.00E-05
PSAT1	2.6.1.52	phosphoserine transaminase	R04173	5.00E-05
GOT2	2.6.1.1	aspartate transaminase	R00355	0.0009
UPP1	2.4.2.3	uridine phosphorylase	R01876	5.00E-05
PNP	2.4.2.1	purine-nucleoside phosphorylase	R08368	0.00295
C1GALT1	2.4.1.122	D-galactosyltransferase	R05908	5.00E-05
DGAT2	2.3.1.20	diglyceride acyltransferase	R02251	0.0002
TALDO1	2.2.1.2	formaldehyde transketolase	R08575	0.0015
PYCR1	1.5.1.2	proline oxidase	R01248	5.00E-05
MTHFD2	1.5.1.15	methylenetetrahydrofolate dehydrogenase (NAD+)	R01218	5.00E-05
DHCR7	1.3.1.21	7-dehydrocholesterol reductase	R01456	0.0012
SCD	1.14.19.1	stearoyl-CoA 9-desaturase	R02222	5.00E-05
LPO	1.11.1.7	peroxidase	R03344	5.00E-05
PGD	1.1.1.44	phosphogluconate dehydrogenase	R03532	0.0022
DCXR	1.1.1.10	L-xylulose reductase	R01904	0.00155

**Table 6:** General properties of the constructed sub-network.

Parameters	Values
Number of nodes	832
Number of edges	1021
Network density	0.003
Network heterogeneity	0.733
Number of self-loops	0
Network diameter	28
Network centralisation	0.15
Average path length	6.913

**Table 7:** First 15 hub metabolites of the acidosis metabolic network.

Metabolites	Neutral Formula	KeggID	PubChemID	Compartment
Stearoyl-CoA	C39H70N7O17P3S	C00412	3702	Endoplasmic Reticulum
Ferrocytochrome b5	C36H61O11	C00996	4242	Membrane
glutathione	C10H17N3O6S	C00051	3353	Cytoplasm
2-oxoglutarate	C5H6O5	C00026	3328	Mitochondrion
protein-L-arginine	C7H13N5O2R2	C00613	3887	Nucleus
electron-transferring flavoprotein	C27H30N9O15P2	C04253	6918	Mitochondrion
ubiquinone	C19H26O4	C00399	5280346	Mitochondrion
S-adenosyl-L-methionine	C15H22N6O5S	C00019	34755	Cytoplasm
S-adenosyl-L-homocysteine	C14H20N6O5S	C00021	439155	Nucleus
Acetyl-CoA	C23H38N7O17P3S	C00024	3326	Cytoplasm
L-tryptophan	C11H12N2O2	C00078	3378	Cytoplasm
L-arginine	C7H13N5O2R2	C00613	3887	Mitochondrion
L-serine	C3H7NO3	C00065	3365	Cytoplasm
L-Lactate	C3H6O3	C00186	3486	Cytoplasm
Xanthine	C10H13N4O9P	C00655	73323	Nucleus

**Table 8:** Most of the pathways enriched by up-regulated and down-regulated enzymes.

Significant Up-expression enzymes	Fatty acid metabolism	Butyrate metabolism (ec00650) Arachidonic acid metabolism (ec00590) Linoleic acid metabolism (ec00591) Fatty acid degradation (ec00071) Pantothenate and CoA biosynthesis (ec00770)
	Biosynthesis of amino acids	Valine, leucine and isoleucine metabolism (ec00280) Glycine, serine and threonine metabolism (ec00260)
	Pyruvate and carbon metabolism	Glycolysis/Gluconeogenesis (ec00010) Pentose phosphate (ec00030) Pyruvate metabolism (ec00620) Glutathione metabolism (ec00480) Fructose and mannose metabolism (ec00051) Galactose metabolism (ec00052) Synthesis and degradation of ketone bodies (ec00072) Pentose and glucuronate interconversions (ec00040)
Significant Down-expression enzymes	Nucleotide metabolism	Purine metabolism (ec00230) Pyrimidine metabolism (ec00240)
	Other metabolisms	Vitamin B6 metabolism (ec00750) Biosynthesis of antibiotics (ec01130)

## Supplementary Materials

The following are available online at https://www.mdpi.com/2076-2615/10/3/469/s1 file, Table S1: Total of genes expressed in the rumen tissue (UniGene database). Table S2: Fold change, Gene name, primer sequences and product length of qRT-PCR validated genes. Table S3: Primary metabolic network data. Table S4: Statical result of RNA-Seq data analysis. Table S5: The result of detected differentially expressed genes (DEGs) with the RNA-seq data analysis using Tuxedo package. Table S6: Enrichment analysis using KEGG, SP_PIR and GO. Reported p-values was corrected for multiple testing using the Benjamini-Hochberg procedure, enrichment analysis was performed using DAVID with default parameters. Table S7: Acidosis metabolic network data.
